# Incomplete concerted evolution and reproductive isolation at the rDNA locus uncovers nine cryptic species within *Anopheles longirostris *from Papua New Guinea

**DOI:** 10.1186/1471-2148-10-392

**Published:** 2010-12-24

**Authors:** David E Alquezar, Stephane Hemmerter, Robert D Cooper, Nigel W Beebe

**Affiliations:** 1 Institute for the Biotechnology of Infectious Disease, University of Technology, Sydney. Australia; 2 Australian Army Malaria Institute, Brisbane, Australia; 3 School of Biological Sciences, University of Queensland, St Lucia, Queensland, 4072, Australia; 4 CSIRO Ecosystem Sciences, EcoSciences Precinct, Dutton Park, Queensland, 4102, Australia

## Abstract

**Background:**

Nuclear ribosomal DNA (rDNA) genes and transcribed spacers are highly utilized as taxonomic markers in metazoans despite the lack of a cohesive understanding of their evolution. Here we follow the evolution of the rDNA second internal transcribed spacer (ITS2) and the mitochondrial DNA cytochrome oxidase I subunit in the malaria mosquito *Anopheles longirostris *from Papua New Guinea (PNG). This morphospecies inhabits a variety of ecological environments indicating that it may comprise a complex of morphologically indistinguishable species. Using collections from over 70 sites in PNG, the mtDNA was assessed via direct DNA sequencing while the ITS2 was assessed at three levels - crude sequence variation through restriction digest, intragenomic copy variant organisation (homogenisation) through heteroduplex analysis and DNA sequencing via cloning.

**Results:**

Genetic evaluation of over 300 individuals revealed that *A. longirostris *comprises eight ITS2 PCR-RFLP genotypes and nine ITS2 heteroduplex genotypes showing distinct copy variant organization profiles after PCR amplification. Seven of these nine genotypes were found to be sympatric with other genotypes. Phylogenetic analysis of cloned ITS2 PCR products and mtDNA COI confirmed all nine clades with evidence of reproductive isolation at the rDNA locus. Compensatory base changes in the ITS2 secondary structure or in pseudoknots were absent when closely related species were assessed. Individuals from each ITS2 genotype showed the same copy variant heteroduplex profile suggesting that the rDNA array is fixed within each genotype.

**Conclusion:**

The centromere-proximal position of the rDNA array in *Anopheles *mosquitoes has probably reduced interchromosomal recombination leaving intrachromosomal events responsible for the observed pattern of concerted evolution we see in these mosquitoes. The stability of these intragenomic ITS2 copy variants within individuals and interbreeding populations suggests that rDNA is moving as a single evolutionary unit through natural populations to fixation and has provided a complementary diagnostic tool to the restriction digest for studying genetic discontinuities and species boundaries. In this, the utility of the ITS2 as a universal taxonomic marker is probably contingent on several factors pertaining to spacer dimensions and the genomic location of the rDNA array with respect to recombination and proximity to regions potentially under selection.

## Background

The identification and classification of reproductive isolation is fundamental for defining species biodiversity as species are generally understood to be composed of genetically or reproductively isolated units [1]. Two very differently evolving genomic regions are often used to study species diversity and include a segment of the mitochondrial DNA (mtDNA) cytochrome oxidase subunit I (COI) gene and the ribosomal DNA (rDNA) second internal transcribed spacer (ITS2) [2,3]. The only commonality between these markers is that both regions exist in high copy numbers within eukaryotes, which in turn facilitates their PCR amplification. The conditions under which they evolve could not be more different.

The use of a barcoding approach based on mitochondrial DNA (mtDNA) provides a line of evidence from a genome separate to nuclear DNA in that it is not associated with the machinery of sex and also carries several intrinsic problems associated with its evolution including maternal inheritance, reduced effective population size, introgression and heteroplasmy [4]. Importantly, when studying closely related species the mtDNA is often unable to identify recently emerged species because of the time required to separate the intraspecific variation from interspecific divergence - the "barcoding gap" [4,5]. Indeed, barcoding gap problems can occur in discriminating species within Diptera [6]. At the same time, the ITS2 has been used extensively as a tool for the identification of species with increasing calls for this region to become an important marker in molecular systematics based on the occurrence of a correlation between compensatory nucleotide changes on helix II or III of the ITS2 secondary structure and sexual incompatibility [3,7,8].

In metazoans, the ITS2 is part of the rDNA gene family tandemly organized head to tail, often hundreds of times in the nucleolar organizer regions (NORs). This family of structural RNA genes and spacers are observed to evolve through a pattern of concerted evolution using DNA turnover machinery that operates to spread or remove sequence variant copies within individuals (homogenization), and also operates to spread or remove variants within an interbreeding population (fixation). Polymorphism is usually low among these tandem units within individual genomes and populations due to the homogenizing effect of the DNA turnover machinery. Unlike the mtDNA barcode marker, whose evolution and utility as a taxonomic marker is relatively well described [9,10], the evolutionary machinery that drives variation in nuclear rDNA is more complex - being nuclear DNA that does not follow traditional Mendelian rules of inheritance - and a comprehensive understanding of this process remains elusive [11-13]. However, in metazoans we observe that each gene sequence in the array is generally the same, producing sequence similarity within a species and sequence diversity between species [14,15]. The practicality of this rDNA turnover machinery for resolving closely related species has been that the less functionally restricted regions, including the ITS2, can accumulate mutations within reproductively isolated populations relatively quickly and can thus be the first indicators of genetic discontinuity between populations [3,7,16]

Mosquitoes transmit pathogens that cause human disease. As each species will display its own biology, ecology and pathogen transmission potential, identification of independently evolving genetic groups is vital to an understanding of mosquito-borne disease dynamics. It is generally understood that many mosquito morphospecies contain cryptic species, and the occurrence of newly identified species is usually followed by the development of molecular diagnostic tools to assist field studies [17,18]. These diagnostic tools are commonly designed around the fast evolving ITS2, which have consistently revealed informative species-level sequence variation and are flanked by conserved gene regions, greatly simplifying PCR primer design and analysis.

The mosquito *Anopheles longirostris *transmits malaria in Papua New Guinea (PNG) [19,20]. This morphospecies exists throughout the major river valleys and flood plains of the Sepik and Ramu valleys in northern PNG, as well as the upper Fly River valley in southwest PNG; it inhabits a variety of ecological environments indicating that it may comprise a complex of morphologically indistinguishable species [21]. The aim of this study is to determine if *A. longirostris *constitutes a cryptic species group by showing reproductive isolation at the rDNA ITS2 locus, while co-investigation of the COI locus is used to determine if the identified cryptic species exist as divergent mtDNA lineages [5]. We hypothesize that *A. longirostris*, like many mosquito morphospecies in PNG, comprises a cryptic species complex and so we follow the evolution of both the mtDNA COI and rDNA ITS2 sampled from over 70 collection sites. We examine the evolution of the ITS2 at three levels: 1) PCR-restriction fragment length polymorphism (RFLP) to view crude sequence variation; 2) PCR-copy variant analysis to view the homogenization of potential ITS2 copy variants within the rDNA array; and 3) DNA sequence analysis via cloning and sequencing.

Concordance was found between the numerous mtDNA lineages and ITS2 genotypes and reproductive isolation exists at the rDNA locus for most genotypes. Interestingly the ITS2 copy variants appear fixed in all individuals of each genotype suggesting that intrachromosomal events are the primary driver of rDNA turnover in *A. longirostris *and that the rDNA array is moving as a single evolutionary unit within interbreeding populations.

## Results

**Figure 1:**
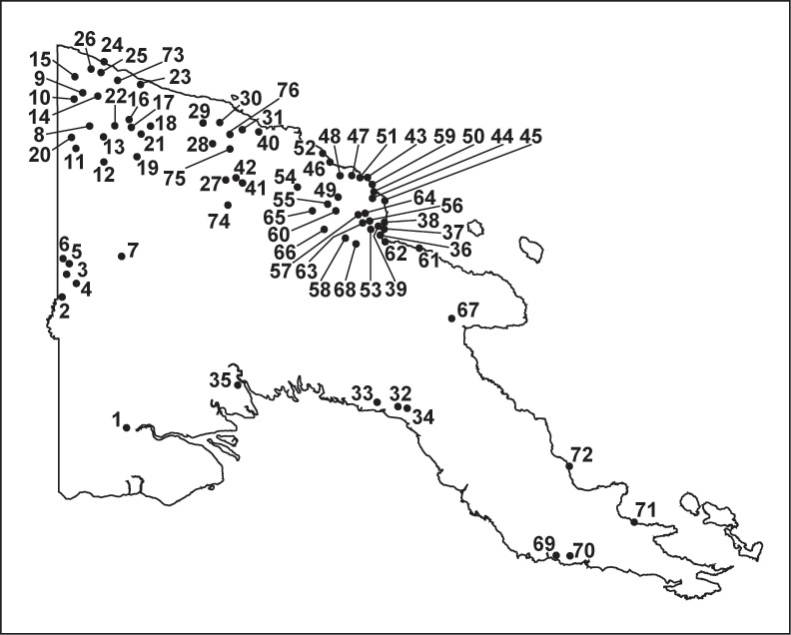
**Map of Papua New Guinea showing collections sites of *A. longirostris***.

**Figure 2:**
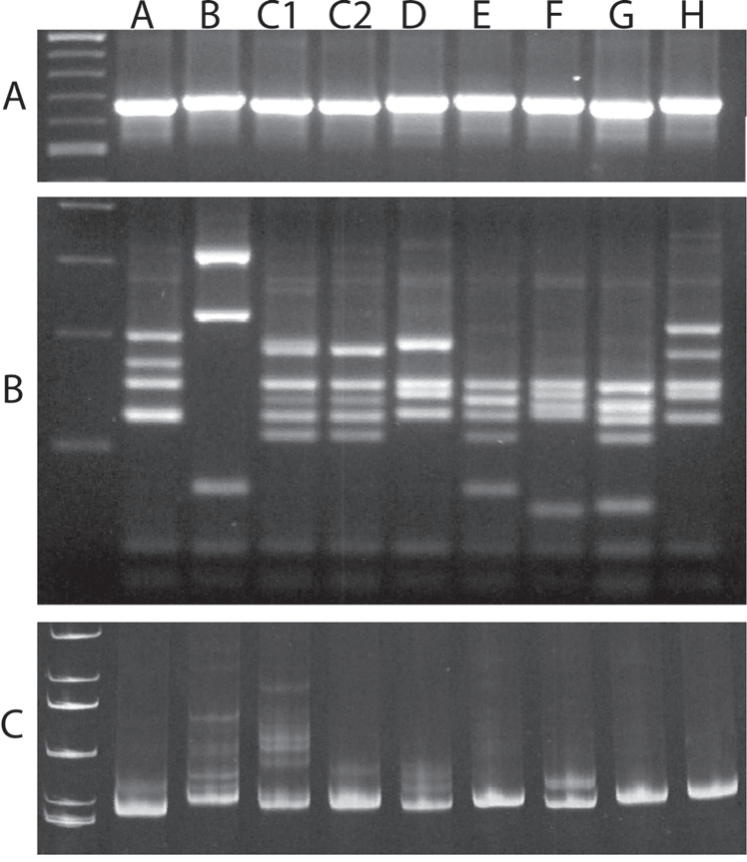
**ITS2 genotypes of *A. longirostris***. Panel A: ITS2 PCR products run through a 1.0% agarose gel indicate some small size variation. Panel B: The same ITS2 PCR products cut with *Msp *I and run through a 3.0% agarose gel reveals eight distinct RFLP genotype profiles. Panel C: Most ITS2-RFLP genotypes revealed the same heteroduplex profile when PCR products were run through a 7.0% acrylamide gel suggesting copy variants are fixed within individuals and within interbreeding populations. However, genotype C contained two distinct heteroduplex profiles (designated C1 and C2) within its RFLP profile revealing the presence of two independently evolving ITS2 genotypes.

A total of 302 specimens identified as *A. longirostris *morphospecies were collected and studied from 76 sites in PNG (Table 1 and Figure 1). Based on PCR amplification of the ITS2 region, all individuals produced a single band at approximately 680 bp (Figure 2A). The ITS2 region was subsequently assessed using three different approaches: 1) rudimentary assessment of sequence variation was obtained by RFLP analysis; 2) intragenomic copy variants present were observed by heteroduplex analysis; and 3) DNA sequence variation was detailed through cloning and sequencing of the ITS2.

**Table 1:** Summary of mosquito collection sites in PNG

Map Site	Year Site	Latitudes	Longitudes	Sample Number*
1	1993/2	-3.82139	141.4575	12
2	1993/3	-3.3483	141.3457	1
3	1993/5	-3.41618	141.2422	12
4	1993/6	-4.14264	141.2604	2
5	1993/8	-4.34685	141.6606	1
6	1993/10	-3.97867	141.654	12
7	1993/14	-3.38709	141.586	14
8	1993/17	-3.10225	141.2592	2
9	1993/22	-3.81907	141.0429	1
10	1993/23	-3.8498	142.061	3
11	1993/27	-3.83852	142.3608	10
12	1993/32	-4.27391	142.1417	1
13	1993/42	-3.96715	141.2027	2
14	1993/43	-3.93283	142.2043	10
15	1993/45	-3.82202	141.8232	2
16	1993/47	-3.20563	142.1934	1
17	1993/49	-2.83613	146.226	1
18	1993/53	-3.04691	141.6254	5
19	1993/54	-2.98728	141.4841	4
20	1993/99	-3.87055	143.7083	5
21	1994/52	-7.94629	145.9866	1
22	1994/53	-7.86491	145.6693	1
23	1994/62	-7.94385	146.1063	1
24	1995/86	-4.55097	145.1288	0 (4)
25	1992/68	-6.10497	141.3719	0 (1)
26	1992/63	-5.99314	141.1222	4
27	1992/79	-5.73255	141.9284	3
28	1992/71	-5.79571	141.0625	3
29	1992/70	-5.11318	141.1066	4
30	1992/62	-6.31728	141.0249	12
31	1992/28	-8.14579	141.983	6
32	1992/65	-6.1281	141.2741	1
33	1994/63	-7.58825	143.687	1
34	1995/1	-5.29123	145.7411	1
35	1995/118	-5.21938	145.5545	1
36	1995/18	-3.89964	143.9252	1
37	1995/34	-4.63884	143.6642	1
38	1995/35	-4.62367	143.6128	1
39	1995/74	-4.61152	145.4955	4
40	1995/75	-4.86588	145.6067	1
41	1995/78	-4.3246	145.0033	1
42	1995/90	-4.60145	145.4423	3
43	1995/98	-4.20768	144.448955	5
44	1995/111	-4.71896	144.4958	3
45	1995/114	-4.98081	144.9456	4
46	1995/120	-5.12045	145.4077	2
47	1995/123	-5.46082	145.2102	1
48	1995/125	-5.56242	146.1766	0 (1)
49	1995/130	-4.78272	145.6285	3
50	1995/134	-5.59874	146.2804	1
51	1995/138	-5.11788	145.4628	2
52	1995/157	-5.07679	144.7181	1
53	1997/122	-10.1185	148.296	1
54	1997/128	-10.1015	148.4675	1
55	1998/171	-9.62046	149.4318	1
56	1998/183	-8.81208	148.4748	1
57	1993/76	-4.06985	143.2562	9 (2)
58	1995/137	-5.23905	145.4598	11 (1)
59	1995/4	-5.29396	145.7483	10 (2)
60	1995/7	-5.28947	145.7628	11 (1)
61	1995/15	-5.4142	145.7264	11 (1)
62	1995/77	-4.91868	145.7815	12
63	1995/88	-4.87637	145.0901	1
64	1995/89	-4.6723	145.5873	12
65	1995/81	-4.56498	145.3099	10 (2)
66	1995/86	-4.55097	145.1288	1
67	1995/106	-4.6723	145.5873	12
68	1995/132	-5.07245	145.0517	4
69	1995/136	-5.48847	145.7741	3 (1)
70	1996/75	-6.57615	146.8209	11
71	1995/158	-5.33051	144.8912	1
72	1996/136	-5.53449	145.3597	1
73	1993/54	-2.98728	141.4841	2
74	1993/70	-4.61974	143.4462	3
75	1993/94	-3.76457	143.1519	1
76	1993/98	-3.78299	143.3662	1

*Numbers in parentheses are mosquitoes morphologically misidentified as *A. longirostris*.

### Analysis of the ITS2 RFLP data

Restriction digestion of the ITS2 product with the enzyme *Msp *I (recognition motif CCGG) followed by size separation through a 3% agarose gel revealed eight RFLP profiles or genotypes with bands ranging from 50 bp to 350 bp. These eight genotypes have been designated A-H (Figure 2B). Genotype distribution and abundance are described in Table 2. Briefly, genotype A was only found from one site in the Ramu Valley north-west PNG; genotype B came from 11 sites both north and south of the PNG Central Ranges; genotype C was common and found at 37 sites throughout PNG; genotype D came from nine sites spanning both sides of Central Ranges; genotype E came from 16 sites throughout PNG; genotype F came from three sites in north-west PNG; genotype G was restricted to sites in Western PNG, on both sides of the Central Ranges; and genotype H came from two sites in north-west PNG. No shared or hybrid RFLP profiles were found and only two RFLP genotypes (F and H) were not found to be in sympatry with the other RFLP genotypes (Table 3), suggesting potential reproductive isolation.

**Table 2:** ITS2 genotype distribution and abundance summary

ITS2 RFLP Genotype	Number of specimens	Frequency (%)	Map site
Genotype A	4	1.3	6
Genotype B	25	8.2	1, 4, 6, 7, 13, 15, 33, 37, 38, 73, 74
Genotype C1	11	3.6	1, 3
Genotype C2	170	56.3	1, 2, 3, 7, 14, 16, 17, 18, 26, 28, 30, 36, 40, 42, 43, 44, 45, 46, 47, 50, 52, 55, 57, 58, 59, 61, 62, 63, 64, 66, 68, 69, 70, 71, 72
Genotype D	24	7.9	3, 5, 7, 18, 19, 30, 32, 57, 75
Genotype E	38	12.1	7, 8, 10, 11, 12, 20, 34, 39, 42, 49, 50, 58, 64, 67, 70, 76
Genotype F	8	2.6	23, 31, 53
Genotype G	17	5.6	7, 9, 11, 27, 29
Genotype H	5	2.6	26, 30
	**Total 302**		

**Table 3:** Sympatric ITS2 genotypes

Collection Site	Sympatric Genotypes
1	B, C1, C2
3	C2, D
5	C1, C2, D
6	A, B
7	B, C1, C2, D E, G
11	C2, E, G
15	B, C2
30	C2, D
57	C, D
58	C, E
64	C, E
67	C2, E
70	C, E

### Identification of ITS2 copy variants from within each genome

To assess the ITS2 homogenization status (i.e. the presence of copy variants within each genome) and inform on the potential for direct DNA sequencing, PCR products were run through a 7.0% native acrylamide gel. Discrete homogenization profiles appeared consistent for seven of the eight RFLP genotypes (Figure 2C; Table 2). However, the RFLP genotype designated C revealed two distinct heteroduplex profiles, suggesting that two different copy variant populations were present within this single RFLP profile, and these were assigned their own genotype status (designated C1 and C2). Thus ITS2 genotypes could be resolved within RFLP profiles by following the copy variant organization in individual genomes. Additionally, seven of the nine genotypes were found to be in sympatry, again suggesting potential reproductive isolation at the rDNA locus (Table 3).

### ITS2 DNA sequence analysis

Three individuals from each of the seven ITS2 genotypes were selected and cloned with four to five clones sequenced per individual. The ITS2 PCR product cloning and DNA sequencing is summarized in Table 4 and all unique ITS2 sequences were submitted to Genbank with accession numbers described in the genetic analysis summary Table 5 [GU170434-GU170538]. Cloned ITS2 sequences were subsequently aligned in Clustal X [22] using gap opening/gap extension values of 20/15. The ITS2 alignment length was 762 nucleotides consisting of 105 sequences of which 543 characters (71%) were constant, 168 characters (22%) were parsimony informative and 51 variable characters (7%) were parsimony uninformative. Gaps or missing data represented 264 sites and the GC content ranged from 59.1-64.5% (61.8% average). A summary of the ITS2 sequencing is detailed in Table 4 and 5 with the ITS2 alignment available as additional file 1 or as a Popset through GenBank [269969773].

**Table 4:** Summary of ITS2 genotype cloning and DNA sequencing

	Individual	Year/Site	Number of clones^#^	Total clones per Genotype
Genotype A	1A6-1	1993/10	5	
	1A6-3	1993/10	5	14
	1A6-4	1993/10	3	
Genotype B	1A4-1	1993/6	5	
	1D9-1	1995/35	4	9
				
Genotype C1	1A1-9	1993/2	5	
	1A1-1	1993/2	4	14
	1A1-7	1993/2	5	
Genotype C2	1A3-10	1993/5	5	
	1A3-7	1993/5	3	13
	1A3-1	1993/5	5	
Genotype D	1G2-1	1993/76	5	
	1A3-2	1993/5	3	12
	1B8-3	1993/53	5	
Genotype E	1B10-3	1993/99	5	
	1B1-1	1993/27	5	10
				
				
Genotype F	1C3-1	1994/62	5	
	1D1-5	1992/28	5	16
	1F4-1	1997/122	5	
Genotype G	1A7-4	1993/14	5	
	1B1-8	1993/27	4	14
	1C7-1	1992/79	5	
Genotype H*	L1C6_1	1992/63		
	L1C6_2	1992/63		
	L1C6_3	1992/63		
				**110**

**^#^**GenBank sequences [GU247052 - GU247114].

*Genotype H showed no copy variant heteroduplex profiles and could be directly sequenced.

**Table 5:** ITS2 genetic analysis summary

ITS2 Genotype	RFLP band sizes (bp)	ITS2 sequence	mtDNA COI haplotypes
A	200	GU170434-GU170447	GU247052-GU247056
	170		
	155		
	125		

B	312	GU170448-GU170456	GU247057-GU247066
	225		
	< 100		

C1	181	GU170457-GU170473	GU247067-GU247072
	140		
	125		
	112		

C2	181	GU170474-GU170483	GU247073-GU247082
	125		
	140		
	112		

D	187	GU170484-GU170495, GU170521	GU247083-GU247091
	156		
	148		
	130		

E	156	GU170496-GU170506	GU247092-GU247098
	142		
	125		
	108		
	< 100		

F	162	GU170507-GU170520	GU247099-GU248002
	149		
	136		
	126		
	< 100		

G	155	GU170522-GU170535	GU247103-GU247111
	136		
	125		
	106		
	< 100		

H	204	*GU170536-GU170538	H1C63 - GU247112-GU247114
	174		
	155		
	149		
	124		

* No copy variants evident in PCR product

**Figure 3:**
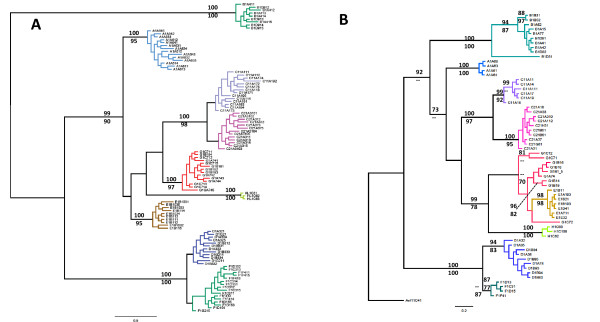
**Phylogenetic assessment of *A. longirostris *based on DNA sequence from the ITS2 (Panel A) and mtDNA COI (Panel B) reveal nine lineages**. Both Bayesian and Maximum Likelihood procedures produced trees of similar topology with branch support values above 70% shown - Bayesian posterior probability above the branch (converted to percentage) and Maximum Likelihood bootstrapping (percentage) below the branch. The ITS2 showed a well resolved tree with all clades well separated into genotypes. All ITS2 genotypes were evident as mtDNA COI divergent lineages with cloned individuals co- assessed for the COI.

Based on NCBI BLAST searches (blastn algorithm), the most closely related sequences came from other regional mosquito species - *A. annulipes *from Australia (EF042773, 74% identity over 100% of the sequence) and *A. lungae *from the Solomon Islands (80% identity over 64% of the sequence). This low level of sequence identity to mosquitoes outside the group led to the construction of unrooted phylogenetic trees using both Bayesian and ML phylogenetic methods and produced similar trees with strong bootstrap scores and posterior probabilities for the nine ITS2 genotypes (Figure 3). All ITS2 sequences were unique for each divergent lineage and no evidence of either hybridization or ancestral polymorphisms was found between the nine lineages.

Intraindividual ITS2 sequence variation revealed by both heteroduplex profiles and in cloned ITS2 DNA sequences appeared as single nucleotide polymorphisms and sequence insertion/deletion indels, where indels generated overt hetroduplex bands that migrated much more slowly through the acrylamide gel. For example, genotypes B, C1, C2, D and F contain ITS2 copy variant sequence indels that manifest in the acrylamide gel as slower moving heteroduplexes (Figure 2). (Refer to the ITS2 alignment in additional file 1 or in GenBank [Popset: 269969773] for indel locations (genotypes B (nt 486-487), C1 (nt 454-455, 612-614, 621-624), C2 (nt 530), D (nts 571, 610, 610-611), and F (nt 533, 570, 573, 577)).

### MtDNA COI DNA phylogenetic analysis

Sixty-two individuals representing each of the nine ITS2 heteroduplex genotypes were sequenced for a segment of the mtDNA COI [GenBank submissions GU247052 - GU247114]; the results are summarized in Table 5 and the alignment is available as additional file 2 and Genbank Popset [82555717]. There was no evidence of intragenomic sequence polymorphisms in the chromatograms to suggest the presence of nuclear mitochondrial sequences or pseudogenes. The final 524 bp alignment contained 41 different haplotypes, consisting of 103 variable sites of which 98 (or 95%) were parsimony informative. All ITS2 lineages were evident in the mtDNA COI. One non-synonymous T to G transversion was identified at residue 221 in all samples of genotype B resulting in a change from Serine to Alanine. The COI data shows well-supported clades for eight of the nine ITS2 genotypes. However the clade for genotype E sits as a subclade with genotype G, separated by 9-10 mutational steps (1.7-1.9%) from the nearest G haplotype. Diversity within genotype G spans 17 mutational steps at its maximum (3.3%) suggesting that without the ITS2 data, genotype E may not have been resolved with the COI alone because it exists in a "barcode gap" between the substantial intraspecific variation of genotype G and the small interspecific divergence of genotype E. More sampling may help resolve this issue.

### ITS2 secondary structure analysis

Other studies have raised the suggestion that compensatory base changes in ITS2 secondary structure can correlate with sexual incompatibility and discriminate of biological species [3,7]. We assessed closely related ITS2 genotypes (C1-C2 and F-D) for the presence of compensatory base changes because they showed distinct genotype-specific sequence indels. ITS2 sequences were folded into secondary structures using the MFOLD algorithm [23], with optimal and suboptimal structures generated and observed for compensatory base changes. Despite the presence of genotype specific indels identified in the 3' region (25 bp separated the F/D comparison and 53 bp separated the C1/C2 comparison), no optimal or suboptimal structures could place these indels in positions that could be observed as compensatory. Additionally, the issue of compensatory base changes within RNA pseudoknots also seemed unlikely but could not be fully explored due to the 450 bp sequence length limits of the vsfold5 pseudoknot prediction software [24]. However, as the ITS2 sequence folded consistently 5' to 3' in MFOLD we could utilize partial sequences in the pseudoknot analyses. Nevertheless, we found no evidence that these nucleotide differences between genotypes were involved or linked to pseudoknots.

### Genotype abundance and distribution in PNG

The abundance and distribution of the nine ITS2 genotypes are described in Table 2. The RFLP genotype C was most commonly collected with 181 specimens representing 60% of the collection. However, subsequent acrylamide gel analysis to reveal heteroduplex copy variants split this RFLP genotype into subsets of 11 C1 (3.6%) and 170 C2 (56.3%). The C1 genotype was only collected from PNG's northern Sepik Valley region while C2 extended across the full distribution of *A. longirostris *- a range also shared by genotypes B, D and E.

## Discussion

The malaria transmitting mosquito identified morphologically as *A. longirostris *was found to comprise nine cryptic species in PNG that could be resolved by following the evolution of two molecular markers - the rDNA ITS2 and the mtDNA COI. The ITS2 is a transcribed spacer comprising part of a nuclear rDNA multicopy gene family and evolves through non-Mendelian inheritance processes [25,26]. By following the evolution of the ITS2 it was possible to show evidence for reproductive isolation at the rDNA locus and also develop relatively simple species-diagnostic molecular tools to distinguish these putative cryptic species. The maternally inherited COI, widely used as a DNA barcode [27], was able to show the existence of eight divergent lineages with two genotypes (G and E) potentially obscured by the substantial intraspecific variation of genotype G and small interspecific divergence of genotype E.

The ITS2 revealed eight overt RFLP profiles with genotype discrimination apparent through single nucleotide polymorphisms and repeat insertion/deletion indels that are common to these regions. Seven of the eight RFLP genotypes generated a single heteroduplex profile with the RFLP of genotype C revealing two distinct heteroduplex profiles and thus suggesting that it contains two distinct ITS2 genotypes. Phylogenetic analysis of the cloned ITS2 and COI sequences from individuals representing each genotype revealed nine separate evolutionary units confirming all genotypes including both C1 and C2; these latter two appear as distinct sister clades. Agreement with the mtDNA COI on these species' groupings provided additional support for the ITS2 data, but this comparative approach may not be as informative for recently diverged species that do not show complete COI lineage sorting.

Despite the incomplete homogenization of the ITS2 in these mosquitoes, the spacer appears to evolve through concerted evolutionary rDNA turnover suggesting that interbreeding in reproductively isolated populations is driving different rDNA patterns that have in turn facilitated the discrimination of multiple species. It seems unlikely that the copy variants observed within these genotypes are the products of rDNA pseudogene amplification as we found little evidence of an elevated mutation rate in the conserved 5.8 S 3' gene region that makes up the first 100 nt of the ITS2 alignment. Additionally, no optimal or suboptimal ITS2 secondary structure folds (including RNA pseudoknots) could identify compensatory base changes when closely related ITS2 genotypes were examined that would have concurred with the theory that compensatory base changes in the ITS2 secondary structure correlate with sexual incompatibility [3,7].

The mitochondrial DNA is regarded as fast evolving, but its mode of maternal inheritance can only reveal the presence of divergent lineages and cannot confirm the existence of reproductive isolation between lineages. Discrimination of species based on a percentage of divergence thresholds has been suggested [27], but this has been questioned for studies of Diptera [6]. The advantage of using the rDNA as a genetic marker is that it is possible to identify reproductive isolation at the rDNA locus where different rDNA genotypes exist in sympatry. Under this assumption, most *A. longirostris *ITS2 genotypes were collected in sympatry with other genotypes (except H and F) while still maintaining genotype-specific RFLP and heteroduplex profiles. Additionally, most genotypes show robust phylogenetic clustering with no shared sequences that could be considered either ancestral or a result of recent hybridization. The mtDNA COI marker supports all nine genotypes with evidence of extended time in isolation in most cases. We suggest that *A. longirostris *is a complex of at least nine cryptic species in PNG that can now be distinguished by a relatively simple PCR-based procedures utilizing the ITS2.

The utility of the ITS2 as a marker that can show early genetic discontinuity between populations may arise from a combination of interlinked factors that include the ITS2 sequence length, the position of the rDNA array on the chromosome with an associated ability to facilitate recombination. The size of the ITS2 appears to have some bearing on the ability of the sequence to acquire non-deleterious mutations with longer sequences generally better able to accommodate mutation accumulation than shorter sequences. If indeed a core structure is necessary for maturation of the rRNA, mutations in a minimum length structure will affect the core structure integrity moreso than longer ITS2 sequences that may accommodate a higher level of mutation. For example, the extension and contractions of helices through the gain and loss of nucleotides and indels may not compromise the structure-function relationship. Using examples from Diptera, short ITS2 sequences such as those found in Nearctic Blackfly species (Diptera: Simuliidae; ITS2 length 286-260 nt) show a restricted number of informative sites when examined across five genera [28]. The ITS2 sequences from members of the *Anopheles gambiae *complex (ITS2 ~ 426 nt) reveal a low level of single nucleotide polymorphisms between cryptic species [16,29]. This study and similar studies on *Anopheles *mosquitoes from PNG suggest some correlation between rDNA transcribed spacer size - whether it be ITS2 or ITS1 - and the level of DNA sequence variation and homogenization, with longer spacers showing a more complex pattern of incomplete concerted evolution and DNA sequence variation that can be informative at the inter- and intraspecific level [11,30-32].

The DNA turnover machinery responsible for this observed pattern of concerted evolution is thought to be driven primarily through unequal crossover and gene conversion [13,33]. If recombination is reduced, as it is near the centromere [34], then homogenization may appear incomplete and copy variants can be carried along within an interbreeding population. The rDNA in *Anopheles *mosquitoes is located on the sex chromosomes adjacent to the centromeres [35], and so rDNA evolution would be driven primarily by intrachromosomal events, most likely gene conversion, within the rDNA array. The presence of fixed ITS2 variants within the *A. longirostris *rDNA genotypes suggests the rDNA array is moving as a single evolutionary unit through interbreeding populations. Comparable studies have come from work on *Drosophila melanogaster*, where the rDNA array exists within paracentromeric heterochromatin on sex chromosomes resulting in reduced recombination that sees rDNA turnover also occurring through intrachromosomal exchanges with the rDNA array evolving as a single evolutionary unit or locus [36]. Intrachromosomal events driving rDNA evolution have also been suggested by others [37,38], and the DNA turnover machinery suggested is gene conversion. Indeed biased gene conversion can be dynamic as seen in hybrid arthropod scallops where experimental hybridisation between closely related scallop species carrying different rDNA ITS variants can induce rapid early development biased gene conversion (to the maternal type) that is almost complete 14 days after fertilization [39].

*Anopheles longirostris *revealed nine independently evolving rDNA genotypes with each genotype showing fixed ITS2 sequence variants and suggesting that the rDNA array is moving as a single evolutionary unit or locus. This observation is evident in other *Anopheles *mosquitoes we have studied and has previously provided insights into genetic subdivisions within and between putative species [30-32]. These regions may be under divergent natural selection as evolutionary studies on *A. gambiae *from Africa see the X-linked centromere-proximal rDNA in a region under reduced gene flow, which has led to the suggestion that suppressed recombination in this region may be involved in speciation [40,41]. The rDNA in *A. gambiae *and other *Anopheles *mosquitoes probably reside in regions that are now regarded as genomic islands of speciation (areas of the genome which show high levels of differentiation early in the divergence process) which often include genes responsible for behavioral or ecological isolation [42,43]. Linkage disequilibrium between the rDNA and potential isolating factors or genes under selection may have contributed to the ITS2 evolution we see in these cryptic species from PNG.

## Conclusion

The rDNA ITS2 is now regarded as a molecular key for the identification of eukaryote biological species [3,7]. However, while the utility of the ITS2 for species identification can be compelling - as seen in this study, amongst many others - it is most probably contingent on transcribed spacer dimensions and the position on the chromosome of the rDNA array itself with respect to recombination frequency and proximity to genomic regions under selection. We suggest that the rDNA's movement as a single evolutionary unit through these mosquito populations is the reason why we observe fixed intragenomic ITS2 variants within individuals and interbreeding populations. This DNA turnover phenomenon that appears to fix copy variants within interbreeding populations provides a complementary tool for identifying genetic discontinuities and species boundaries.

## Methods

### Sample collection

Mosquitoes were collected from the field throughout regions in PNG by the Australian Army Malaria Unit (AMI) using larval collections, CO_2 _baited light traps or night biting catches [21]. Sites are summarized in Table 1 and displayed in Figure 1. Larval material, in most cases, was bred through to adults with subsequent morphological identification to species using the keys of [44]. All field material was stored either at -20°C, in alcohol or dried on silica gel.

### ITS2 amplification and RFLP genotype scoring

Genomic DNA was extracted and the ITS2 was amplified by PCR using whole or partial mosquitoes and the methods described in [30]. Forward and reverse primers were redesigned to anneal at higher temperatures to reduce the effect of strong secondary structure found in this mosquito's ITS2 region (ITS2Ah 5'-GGA TCG ATG AAG ACC GCA GCTA and ITS2Rh 5'-CCG TTT CGC TCG CAG CTA CTC AGG). The PCR was carried out in 48 well (0.2 ml) PCR cycle plates (Astral Scientific) using 25 μl final volume and oil overlay. The final PCR mixture contained 16.6 mM [NH4]_2_SO4, 67 mM Tris-HCl pH 8.8 (at 25°C), 0.45% Triton X-100, 0.2 mg/ml gelatin, 1.5 mM MgCl, 0.2 mM of each dNTP, 0.4 μM of each primer, 5.0% DMSO, 1.0 unit of *Taq *polymerase and approximately 2-10 ng of purified genomic DNA (1 μl of mosquito DNA extraction). A drop of mineral oil was overlaid and the reaction cycling (on either a MJ research PTC200 or a BioRad C-1000 thermal cycler) involved an initial denaturation step at 94°C for 3 min followed by 30 cycles of 94°C for 1 min, 57°C for 1 min, and 72°C for 1 min using minimum transition times. The PCR products were run out on a 1% agarose gel containing 0.5 μg/ml ethidium bromide and visualized at 312 nm. Restriction digestion analysis of the ITS2 product was performed by adding 5 μl of PCR reaction to 5μl of 2× *Msp *I buffer containing 1 unit of *Msp *I per reaction. Reactions were incubated at 37°C for 2.0 hr and then run on a 3% agarose gel containing 0.5 μg/ml ethidium bromide and visualized at 312 nm. Genotype profiles were scored.

### ITS2 copy variant analysis and DNA sequencing

One tenth (2.5 μl) of the ITS2 PCR product was electrophoresed on a 7% non-denaturing acrylamide gel (NOVEX or BioRad) for 2.5 hr at 200 V. To prevent heating of the gel and subsequent denaturing of the DNA duplex the gel tank was placed in a plastic container containing an ice and water mixture. The gel was stained with ethidium bromide (5μg/ml) for 1 min and then rinsed twice and visualized at 312 nm. Native acrylamide gels are sensitive to double stranded duplex formation with heteroduplexes (duplex misspairing) running slower than homoduplexes (no misspairing) permitting a qualitative assessment of the major ITS2 copy variants amplified in the ITS2 PCR products. Individuals that produced only homoduplexes (a single band corresponding to the size of the PCR product) could be directly sequenced. Heteroduplexes appearing as multiple band profiles in the acrylamide gel signified the presence of ITS2 copy variants in the PCR product and thus cloning was required prior to DNA sequencing. Briefly, ITS2 PCR products were ligated into the pGEMT vector and transformed into *E. coli *(DH5α) according to the manufacturer's recommendations (Promega, Madison, USA). Positive colonies (white/pale-blue) were stabbed with a sterile pipette tip, briefly immersed into a 0.5 ml PCR tube containing a 25 μl PCR reaction, and amplified using both forward and reverse primers as described above. Cycling involved 25 cycles at 94°C for 1 min, 57°C for 1 min, and 72°C for 1 min using minimal transition times. Amplified PCR products were confirmed and quantified on a 1% agarose gel. Sequencing involving a spin column cleanup of amplification products was performed using a QIAGEN QIAquick PCR Purification Kit, (Qiagen, Hilden, Germany) and sequencing was performed by the Australian Genomic Research Facility (University of Queensland) using the ABI Big DyeTM Terminator kit (Perkin Elmer, Forster, USA) according to the manufacturer's recommendations. The same forward and reverse primers were used as a priming template.

### Amplification of the Mitochondrial DNA

A 580 base pair fragment of the mtDNA COI subunit gene was amplified by PCR using the COI forward primer (5' GTTCCTTTAATATTAGGAGCACC 3' [45]) and reverse primer (5' TAATATAGCATAAATTATTCC [46]). The final PCR mixture contained 1× *Taq *buffer II (Fisher Biotech, Australia), 2.5 mM MgCl, 0.125 mM of each dNTP, 0.4 μM of each primer, 0.5-1.0 unit of *Taq *polymerase and 5.0-10.0 ng of extracted genomic DNA (1 μl of extraction). The cycling involved an initial denaturation step of 94°C for 3 min, then 35 cycles of 94°C for 1 min, 50°C for 1 min and 72°C for 1 min with minimal transition times. Amplified PCR products were purified and sequenced as described above.

### Sequence alignment

All sequences were analyzed and edited using Sequencher 4.2.2 (Gene Codes Corporation, 2004) and alignments were generated using Clustal X [22] and edited in MacClade 4 [47]. As the ITS2 regions contained many short repeats and gaps, the final alignment required some manual fine-scale modifications. The ITS2 sequence alignment is available as additional file 1 and as GenBank Popset [269969773].

### Nucleotide substitution model and phylogenetic analyses

The model of molecular evolution for the data was evaluated using jModeltest [48]. Under the Akaike information criterion (AIC), from 88 possible models the ITS2 alignment was best fitted by the GTR+ I followed by the GTR+ Γ model while the mtDNA COI data was best fitted by the TVM+G followed by the GTR+ Γ.

For the ITS2 the nucleotide sequence alignment was analyzed using a Maximum Likelihood method with the model incorporating six categories and a Gamma shape parameter (the GTR+Γ model) using PhyML 3 [49]. The branch support of the Maximum Likelihood tree was evaluated by the bootstrapping method with 500 replicates in PhyML. In addition, the ITS2 dataset was analyzed by Bayesian phylogenetic analysis using MrBayes 3.1.2 [50] using the GTR+ Γ model. Metropolis coupled Markov chain analyses were run with one cold and three heated chains (temperature set to 0.2) for 5,000,000 generations and sampled every 200 generations. This process was performed three times from a random starting tree and run well beyond convergence. Trees before convergence were discarded before reconstruction of the consensus Bayesian tree with posterior probability transformed to percentage values.

The mtDNA sequence was analyzed using a Maximum Likelihood method with the model incorporating six categories and a Gamma shape parameter (GTR+Γ model) using PhyML 3 [49]. The branch support of the Maximum Likelihood tree was evaluated by the bootstrapping method with 500 replicates in PhyML. In addition, the COI dataset was analyzed by Bayesian phylogenetic analysis using MrBayes 3.1.2 [50], using the GTR+ Γ model. Metropolis coupled Markov chain analyses were run with one cold and three heated chains (temperature set to 0.1) for 5,000,000 generations and sampled every 200 generations. This process was performed three times from a random starting tree and run well beyond convergence. Trees before convergence were discarded before reconstruction of the consensus Bayesian tree with posterior probability transformed to percentage values.

### Detection of compensatory substitutions in the ITS2

To investigate the concept of compensatory base changes informing on the potential level of sexual incompatibility [3,7], closely related ITS2 genotypes (C1-C2 and F-D) were assessed for evidence of compensatory base changes. The ITS2 sequences were first folded into secondary structures using the MFOLD algorithm [23]. We used the older MFOLD version 2.3 so as to select for a lower folding temperature of 28°C while leaving other RNA fold parameters as default. Both optimal and suboptimal structures were generated and observed for compensatory base changes. Additionally, the issue of compensatory base changes within RNA pseudoknots was also investigated using vsfold5 pseudoknot prediction software [24]. Limitations in the software (maximum sequence length is 450 bp) only permitted the analysis of partial ITS2 sequences and these were derived from nucleotide 240 to the 3' end based on the folding pattern obtained by MFOLD.

## Authors' contributions

DA performed the experimentation and contributed to writing the manuscript. RDC provided the mosquito material and SH contributed to the phylogenetic analyses. NWB designed the study, contributed to the analyses and wrote the manuscript.

## Supplementary Material

Additional file 1 **S1_ ITS2_A_longirostris**. (ITS2 DNA sequence alignment in Phylip format). Click here for file

Additional file 2 **S2_ COI_A_longirostris**. (mtDNA sequence alignment in Phylip format). Click here for file
